# Application of the AMLprofiler Diagnostic Microarray in the South African Setting

**DOI:** 10.1155/2017/2560191

**Published:** 2017-11-07

**Authors:** S. S. Kappala, M. Alessandrini, T. Matlhako, E. Beltchev, R. Pool, M. S. Pepper

**Affiliations:** ^1^Institute for Cellular and Molecular Medicine, Department of Immunology, Faculty of Health Sciences, and SAMRC Extramural Unit for Stem Cell Research and Therapy, University of Pretoria, Pretoria, South Africa; ^2^Department of Haematology, Faculty of Health Sciences, University of Pretoria and National Health Laboratory Services, Pretoria, South Africa

## Abstract

Acute myeloid leukemia (AML) is characterized by proliferation of the myeloid lineage and accumulation of immature hematopoietic cells in the bone marrow and is typified by marked heterogeneity both in response to treatment and survival. AMLprofiler is a qualitative *in vitro* diagnostic microarray incorporating seven molecular biomarkers used to diagnose and predict posttherapy survival rates. In this study, we compared AMLprofiler to routine AML diagnostic methodologies employed in South Africa, focusing on consistency of the results, cost, and time to result. RNA was isolated from bone marrow and peripheral blood samples from patients with de novo AML and was processed using Affymetrix Gene Profiling Reagent kits. The results from AMLprofiler and standard methodologies were highly comparable. In addition, many samples were determined to be positive for biomarkers not routinely investigated in South Africa, namely, *CEBPA* double mutants, *NPM1* variants, and altered expression levels of *BAALC* and *EVI1*. 38% of samples presented with no positive biomarker; AMLprofiler nonetheless enabled 26% of AML patients to be classified into either favorable or poor prognostic categories. This study highlights the comprehensive nature of the microarray. Decreased time to result and refinement of risk stratification are notable benefits.

## 1. Introduction

Acute myeloid leukemia (AML) is a heterogeneous disorder both biologically and clinically, characterized by accumulation of immature hematopoietic cells in the bone marrow [1]. These cells are commonly referred to as leukemic blasts and lack the capacity to self-renew. Clinical symptoms include fever, fatigue, and spontaneous mucosal and cutaneous bleeding. The leading cause of death is bone marrow failure that results in anaemia, neutropenia, and thrombocytopenia [2]. Diagnosis of AML involves a combination of morphological, cytochemical, and immunophenotyping techniques. Conventional cytogenetic analysis constitutes a crucial part of the standard diagnostic workup for AML patients, as about 55% have chromosomal abnormalities [3].

The World Health Organization (WHO) classifies AML into four major groups based on clinical, morphological, immunophenotypic, and genetic features: (1) AML with recurrent chromosomal abnormalities; (2) therapy-related AML; (3) myelodysplastic syndrome- (MDS-) associated AML; and (4) AML not otherwise specified (NOS) [4]. Prognostic classification is based on data derived from cytogenetics, fluorescent in situ hybridization (FISH), and molecular biology techniques. The karyotype of AML patients is by far the most important prognostic parameter considered when deciding on a specific treatment regime [5]. The prognostic classification of AML patients includes three categories, namely, favorable, intermediate, and poor risk. Approximately 45% of AML patients, who are cytogenetically normal (CN-AML), fall into the intermediate prognostic risk category and are often the most challenging to treat. Prognostic classification is a crucial step in the diagnosis of AML, particularly with respect to identifying those at high risk of relapse and also for category-specific treatment options.

In recent years, the laboratory diagnosis of AML has improved significantly. Microarray technologies, both DNA and gene expression based, are being utilized increasingly for the detection of mutations and changes in gene expression profiles. Additionally, next-generation sequencing technologies are constantly being improved and are also being applied more frequently in this context [6]. Yet, cytogenetic karotyping still remains the gold standard in routine diagnostic procedures for AML patients, which is time-consuming and labor-intensive. Current modalities for AML diagnosis include cytogenetics and molecular approaches (gene mutations and levels of gene expression) which encompasses a wide range of tests when performed individually. The AMLprofiler (SkylineDx, Rotterdam, The Netherlands) is a qualitative *in vitro* diagnostic microarray that uses RNA chemistry to identify seven key molecular biomarkers used for the diagnosis and prognosis of AML: inv(16)/t(16;16), t(8;21), and t(15;17); biallelic *CEBPA* mutation (*CEBPA*dm); ABD-type *NPM1* mutations; *BAALC*-low and *EVI1*-high mRNA expression levels. Each of these biomarkers is associated with either a favorable or poor prognosis (Table 1) and has relevance in cases where the samples have also been shown to be devoid of chromosomal aberrations, that is, fall into the category of cytogenetically normal AML (CN-AML).

The AMLprofiler assay thus allows for the standardized assessment of chromosomal aberrations and molecular abnormalities, which are of clinical significance in AML (Table 1). AMLprofiler is aligned with the global best practice for diagnositic tests in AML and encompasses seven molecular variables including gene mutations and gene rearrangements [7]. High levels of expression of several genes including *EVI1*, *BAALC*, *MN1*, and *ERG* have been found to be prognostically relevant in AML [8, 9]. In 2013, Brand et al. successfully standardized and validated *BAALC* and *EVI1* gene expression markers in a cohort of intermediate cytogenetic risk AML patients [10]. Low *BAALC* expression was associated with a favorable prognosis and high expression of *EVI1* with an unfavorable prognosis. These genes have been incorporated into the AMLprofiler as independent prognostic factors.

In 2016, Nomdedéu et al. reported findings from a feasibility study on AMLprofiler for patient risk stratification in a multicentre trial, which also included a preliminary comparison with the conventional approach [11]. They analysed both the cost factor and turnaround time of AMLprofiler and compared the results with those obtained using conventional diagnostic methods. They further compared the standard prognostic stratification versus the AMLprofiler and concluded that both methods provided significant clinical information. Their results indicated that AMLprofiler was no more expensive than a conventional molecular approach and turnaround times were similar for both approaches. Therefore, they concluded that AMLprofiler could be successfully applied for AML diagnosis in Spain, in order to rapidly identify AML patients with a good prognosis.

In this study, we set out to evaluate the feasibility of utilizing the AMLprofiler in the South African context and also to assess the possible added prognostic value relative to standard procedures. We thus aimed to assess the extent of concordance between the results obtained with traditional diagnostic modalities versus AMLprofiler and also determined whether there might be disparities between the AMLprofiler and standard prognostic stratification that include traditional cytogenetic and molecular methods. We also looked at the cost factor and turnaround time required to execute the AMLprofiler test in the current diagnostic set-up in South Africa.

## 2. Methods

### 2.1. Patient Recruitment and Sample Collection

Approval for this study was obtained from the Research Ethics Committee of the Faculty of Health Sciences, University of Pretoria (Ref. number 42/2012). AML patients were recruited via the National Health Laboratory Service (NHLS) at the Universities of Pretoria and the Witwatersrand and from private pathology groups including Ampath Laboratories and Vermaak and Partners Pathologists. Only adult patients diagnosed with de novo AML, based on a blast count of >20%, were included.

### 2.2. RNA Isolation

The samples were received at the Institute for Cellular and Molecular Medicine (ICMM) laboratory at the University of Pretoria, where RNA was isolated within 48 hours of sample collection. Mononuclear cells were first separated using Ficoll histopaque (Sigma-Aldrich), after which RNA was isolated using the Qiagen RNAeasy kit according to the manufacturer's protocol. The quantity of RNA was checked using a Nanodrop spectrophotometer and RNA integrity analysed on Agilent's TapeStation 2200 before continuing further with the assay. Analysis of 16S and 28S RNA peaks was checked using an RNA Integrity Number (RIN) value. Only samples with a RIN value above 7 were selected for the AMLprofiler procedure.

### 2.3. AMLprofiler Assay

**Figure 1:**
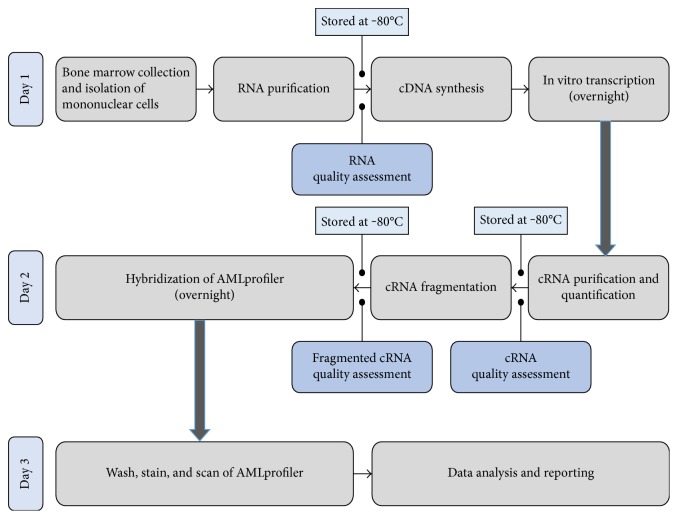
An overview of the AMLprofiler assay. The day to day process flow involved in the AMLprofiler microarray analysis with specific stop points and storage conditions.

The AMLprofiler assay includes different experimental steps that follow in a sequential order and that span over a period of 3 days (Figure 1). This assay was performed according to the manufacturer's protocol. Briefly, the initial step involves synthesis of cDNA from RNA in a two-step reaction that includes first strand and second strand synthesis. The third reaction is an *in vitro* transcription reaction where biotinylated complimentary RNA (cRNA) is synthesized. The cRNA is then purified using magnetic bead separation and analysed using the Agilent TapeStation 2200 to assess for quality and integrity before proceeding to a fragmentation reaction. The cRNA is then fragmented, and the quality was checked again. The final step of the process is the hybridization reaction, where the fragmented cRNA is loaded onto the AMLprofiler microarray and hybridized for 16-17 hours.

### 2.4. Data Analysis and Reporting

Following overnight hybridization, the AMLprofiler microarray was washed, stained, and scanned. After scanning, the data was transferred through a secured centralized server to SkylineDx, The Netherlands, for analysis, and a report was received in the ICMM laboratory within 15–20 minutes.

The results for chromosomal aberrations and gene mutation/expression markers on the AMLprofiler report are displayed as detected/not detected/invalid assay/not applicable. The result shows “detected” in cases where the marker is present, “not detected” in cases where the marker is absent, and “invalid assay” in cases where the data analysis QC parameters do not meet the acceptance criteria. When a patient is detected to be positive for one of the three chromosomal aberrations—inv(16)(p13q22)/t(16;16)(p13;q22), t(8;21)(q22;q22), or t(15;17)(q24;q21)—the patient is classified as being in a cytogenetically “favorable” risk group and is no longer “intermediate” and therefore, the gene expression markers are not applicable in this category. In this case, the results exclusively show “not applicable” on the report. AMLprofiler is designed to detect low expression levels of *BAALC* and high expression levels of *EVI1* genes. The expression marker results are a derivative of an evaluation of the measured expression level of the *EVI1* or the *BAALC* marker against an expression threshold value, so called “cut-off point.” *EVI1* and *BAALC* marker results are currently validated for the “intermediate” cytogenetic risk group only. In case both *BAALC* and *EVI1* show “detected,” it is advised that *EVI1* high “detected” should take precedence over the *BAALC* low “detected.”

## 3. Results

53 adult patients diagnosed with de novo AML (based on a blast count of >20%) were recruited. We analysed 65 AML patient samples which included 49 from bone marrow and 16 from peripheral blood. Matching bone marrow and peripheral blood samples were collected from 12 patients. For this study, we recruited both male (*N* = 35) and female (*N* = 18) AML patients above 18 years of age. Our study included a combination of Caucasian and Black African AML patients from both the public and private health care sectors (Table 2).

When bone marrow samples were assessed, AMLprofiler detected 11 patients with chromosomal aberrations (t(8;21), inv(16)/t(16;16), and/or t(15;17)) and 22 patients with gene mutation and expression markers including *NPM1mut*, *CEBPAdm*, *BAALC-low*, and *EVI1-high*. 20 patients were found to be negative for all cytogenetic and molecular markers present on the AMLprofiler. 14 patients were found to be cytogenetically normal, and AMLprofiler allowed them to be categorized into favorable and poor risk groups. The group with a favorable prognosis included *NPM1* (*N* = 2), *BAALC* (*N* = 6), *NPM1 + BAALC* (*N* = 3), and *CEBPA* (*N* = 1). The categorization of the remaining two individuals into the poor prognosis group was based on increased levels of *EVI1* expression. These two individuals also expressed low-level *BAALC*, which is superseded in the analysis by increased *EVI1* expression. Supplementary Table 1 available online at https://doi.org/10.1155/2017/2560191 provides all the results for individual patients.

With regard to the frequency of cytogenetic and molecular variables on the AMLprofiler, important differences were detected in some cases between the South African population and global averages (Table 3). These included a lower frequency of *NPM1*ABD variants in the South African group (9.4%) when compared to the global average (32.5%) and a higher frequency of increased *EVI1* expression (18.9%) when compared to the global average (9%). We further detected a slightly lower frequency of *CEBPA* double mutants (1.9%) in our cohort when compared to what has been reported globally (5–14%).

In this study, the cost of standard modalities per patient ranged between USD 129 and USD 1167 (average USD 648). Although not offered as a service in South Africa, it is anticipated that the cost of an AMLprofiler diagnostic would be in the order of USD 1000. It is likely however that this would be reduced as economies of scale come to bear. Turnaround time using standard methods was on average 20 days (ranging from 3 to 40 days) from sample collection. The AMLprofiler test can be executed in three days, and the final results can be provided in less than five days.

Our study also included the following results that were discordant: one of the patients was positive for the *CEBPA*dm mutations in the bone marrow sample, while this was negative in the corresponding peripheral blood sample. This indicates that the patient may have had a sporadic *CEBPA*-associated AML where the *CEBPA* mutations were acquired during the course of leukemogenesis [12]. One patient was found to have low expression of *BAALC* in the peripheral blood while being negative in the bone marrow, while another patient was positive for chromosomal aberration t(15;17) only in the bone marrow sample. These BM and PB discrepancies revealed by the AMLprofiler could not be compared to their corresponding routine test results as the latter were not requested in the course of the routine management of these patients. In addition, we detected both t(8;21), t(15;17), and inv(16)/t(16;16) chromosomal aberrations with the AMLprofiler which are not requested in the standard modalities undertaken for AML patients in South Africa. However, our results suggest that AMLprofiler has the potential to change the prognostic stratification of AML patients who do not have major cytogenetic abnormalities, particularly since some of the tests are not currently requested or performed.

## 4. Discussion

In this study, we undertook a microarray-based assessment of molecular variables in AML including chromosomal aberrations, gene mutations, and alterations in gene expression, which offers high sensitivity and specificity and several advantages over diagnostic testing methods currently used. We also evaluated the cost factor and the turnaround time for performing diagnosis with both conventional methods and the AMLprofiler.

Although *NPM1* mutations are the most frequent mutations detected in AML, occurring in 25%–30% of patients [13], in our study, the frequency of *NPM1* mutations was low when compared to the global average. This suggests that the gene mutations assessed for on the microarray might be population specific and are therefore not being detected. In 2014, Marshall et al. reported a lower frequency of known *NPM1* and *FLT3*-ITD mutations in a South African AML cohort and suggested that race-specific mutations might contribute to AML pathogenesis and also to the lower frequency of detection of these mutations [14]. They further concluded that *NPM1* mutation frequency increases with population age and that their cohort had a considerably younger median age at diagnosis, which might contribute to the lower frequency of *NPM1* mutations observed in their study. A similar age-dependent increase in the incidence of *NPM1* mutations has previously been reported [15, 16]. AMLprofiler does not investigate non-ABD mutations within the *NPM1* gene, which indicates that other *NPM1* mutations might be present in the South African population that could not be detected through this method.

AMLprofiler detects high levels of *EVI1* expression in AML patients, which is a poor prognostic marker. Our results show a higher frequency of *EVI1* gene expression compared to other studies that have reported high *EVI1* expression [17]. Furthermore, AMLprofiler is designed to detect low expression of *BAALC*, which is seen as a favorable prognostic marker. The majority of global studies have reported a high expression of *BAALC*, which is associated with an unfavorable prognosis [9, 18]. However, in 2016, Nomdedéu et al. reported a 26% frequency of low *BAALC* expression in their AML cohort [11]. Therefore, only limited comparisons are possible with our *BAALC* results and only from a low expression point of view. The reason for the major difference in the variant numbers of *NPM1* gene mutations between AMLprofiler and standard modalities in South Africa is likely to be due to the fact that the *NPM1* mutation test is not routinely requested by clinicians in South Africa. In addition, neither *CEBPA* nor *BAALC* tests are offered in the South African diagnostic setting.

Further investigations should be performed on larger cohorts of AML samples in the South African population to generate more information and to confirm our results using the AMLprofiler. Samples that are negative for molecular markers should undergo sequencing for the detection of other common biologically relevant mutations in AML including *FLT3*, *KIT*, *RUNX1*, *DNMT3A*, *IDH1*, *TET2*, and *ASXL1*. Next-generation sequencing (NGS) could possibly identify novel genetic markers in a population specific group and add further value to the results generated by AMLprofiler [19, 20]. Further investigation of all the *NPM1*-negative cases using targeted sequencing could possibly identify non-ABD mutations that are common and specific to the South African population. Furthermore, incorporation of other molecular variables of prognostic value on the AMLprofiler might offer an advantage over the current version of the microarray.

Our data revealed that there are both advantages and limitations to using the AMLprofiler. First, AMLprofiler provides for a quicker genetic subtyping of AML and allows for better prognostic stratification of AML patients. An additional benefit is the comprehensive reporting with a quick turnaround time. While routine laboratory diagnostic test results usually take anywhere between 3 and 4 weeks and sometimes even longer, AMLprofiler significantly reduces time between sampling and diagnosis by providing results in 3 days and hence is much quicker in terms of reporting. Since AML progresses rapidly, diagnosis and prognosis play a crucial role in the treatment of these patients. AMLprofiler allows quicker diagnosis as well as prognostic stratification which translates into earlier treatment decisions and also provides valuable time for finding suitable stem cell donors, if applicable. Hence, AMLprofiler enables the clinician to provide the right treatment for AML patients especially in cases which fall into the intermediate risk group with no major chromosomal aberrations.

Furthermore, due to the full standardization of the test, standardized and comparable results can be generated at any diagnostic lab. The costs of implementing and running an AMLprofiler test are also significantly lower when compared to the costs of developing and validating an in-house set of tests in each diagnostic lab. Routine clinical diagnostic methods for AML in South Africa involve cytogenetic testing, FISH, karyotyping, and PCR for detection of chromosomal aberrations and molecular variants which often are time-consuming and tedious laboratory procedures. AMLprofiler replaces seven separate assays including 3 chromosomal aberrations, 2 gene mutations, and 2 gene expression changes in one single test, which saves a considerable amount of time and labor. Hence, AMLprofiler covers a wide range of molecular markers that are used for standard AML diagnosis since it presents a single platform for detection of seven diagnostic markers. AMLprofiler has an added benefit in terms of high specificity and sensitivity with gene expression markers as it is designed to detect low levels of *BAALC* expression and high levels of *EVI1* expression.

Notwithstanding the benefits provided by the AMLprofiler, the absence on this microarray of mutations in the *FLT3* gene, which is a crucial prognostic marker for AML, is a significant limitation. Therefore, a fully comprehensive diagnosis will require a separate investigation of *FLT3* variants. In addition, the AMLprofiler does not report non-ABD mutations in the *NPM1* gene. Finally, the AMLprofiler requires a sophisticated platform for processing that is not common in diagnostic laboratories in South Africa.

## 5. Conclusion

Although microarray technology is not being utilized routinely for AML diagnosis in the South African clinical setting, it might prove to be an important tool to categorize AML patients into prognostic risk groups and thereby assist clinicians to provide tailored therapy for these patients. AMLprofiler offers a more comprehensive investigation of AML samples, adds significant prognostic value, and decreases time to result. AMLprofiler also offers consistent pricing when compared to standard modalities in the current setting in South Africa, and this is likely to be reduced when economies of scale come to bear. We therefore conclude that the AMLproflier would be a useful tool for AML diagnosis and patient stratification in the South African clinical setting.

## Supplementary Material

Supplementary Table 1: AMLprofiler cytogenetic and molecular marker results from individual patient samples.

## Figures and Tables

**Table 1:** Chromosomal aberrations and molecular marker variants detected by AMLprofiler.

Markers on AMLprofiler		Prognostic category
Chromosomal aberrations	inv(16)(p13q22)/t(16;16)(p13;q22)	Favorable
	t(8;21)(q22;q22)	Favorable
	t(15;17)(q24;q21)	Favorable

Gene mutations	*CEBPA* double mutant	Favorable
	*NPM1*-ABD mutations	Favorable

Gene expression	*EVI1*-high expression	Unfavorable
	*BAALC*-low expression	Favorable

**Table 2:** Sample characteristics.

Sample characteristic	Value
Number of study participants	53
Number of samples analysed	65
Source of sample	
Bone marrow (BM)	49
Peripheral blood	16 (with 12 matching BM)
Gender	
Male	35 (66%)
Female	18 (34%)
Race distribution	
Caucasian	20 (37.7%)
Black African	33 (62.3%)
Health sector	
Public	35 (66%)
Private	18 (34%)
Age distribution	44 ± 17.4
Blast count	55% ± 26%

Age distribution and blast count are expressed as mean ± standard deviation.

**Table 3:** Variant frequency of molecular markers on AMLproflier.

Molecular markers on AMLprofiler	Frequency in our current study	Global frequency in AML	Frequency in Black Africans (*n* = 33)	Frequency in Caucasians (*n* = 20)	References
inv(16)(p13q22)/t(16;16)(p13;q22)	5.7%	5–10%	1.9%	3.8%	[5, 21–27]
t(8;21)(q22;q22)	11.3%	7%–10%	5.6%	5.7%	[5, 26–30]
t(15;17)(q24;q21)	3.8%	5–13%	1.9%	1.9%	[24, 26, 27, 29, 31–33]
*CEBPA* double mutant	1.9%	5–14%	0	1.9%	[34–41]
*NPM1*-ABD mutations	9.4%	25–40%	1.9%	7.5%	[13, 16, 42–47]
*EVI1*-high expression	18.9%	8–10%	15.1%	3.8%	[10, 17, 48–50]
*BAALC*-low expression	28.3%	26%	13.2%	15.1%	[11]
