# Supplementary material for: Application of the AMLprofiler Diagnostic Microarray in the South African Setting
Source: Stem Cells Int. 2017 Nov 7;2017:2560191. doi: 10.1155/2017/2560191 (PMC5697127; doi:10.1155/2017/2560191)
Supplement: Supplementary file 1 — Supplementary Table 1: AMLprofiler cytogenetic and molecular marker results from individual patient samples. [file 2560191.f1.pdf]

Supplementary Table 1

AMLprofiler cytogenetic and molecular marker results from individual patient samples

Sample ID	Inv (16)	t(8;21)	t(15;17)	CEBPA	NPM1	BAALC	EVI1
1a	Not detected	Not detected	Not detected	Not detected	Not detected	**Detected**	Not detected
2a	Not detected	Not detected	Not detected	Not detected	Not detected	Not detected	Not detected
3a	Not detected	Not detected	Not detected	Not detected	Not detected	Not detected	Not detected
**4a**	Not detected	Not detected	Not detected	Not detected	Not detected	Not detected	Not detected
**4b**	Not detected	Not detected	Not detected	Not detected	Not detected	Not detected	Not detected
**5a**	Not detected	Not detected	Not detected	**Detected**	Not detected	Not detected	Not detected
**5b**	Not detected	Not detected	Not detected	Not detected	Not detected	Not detected	Not detected
**6a**	Not detected	Not detected	**Detected**	Not detected	Not detected	Not applicable	Not applicable
**6b**	Not detected	Not detected	**Detected**	Not detected	Not detected	Not applicable	Not applicable
7b	Not detected	Not detected	Not detected	Not detected	Not detected	Not detected	Not detected
8a	Not detected	**Detected**	Not detected	Not detected	Not detected	Not applicable	Not applicable
9b	Not detected	Not detected	Not detected	Not detected	Not detected	Not detected	Not detected
**10a**	Not detected	**Detected**	Not detected	Not detected	Not detected	Not applicable	Not applicable
**10b**	Not detected	**Detected**	Not detected	Not detected	Not detected	Not applicable	Not applicable
11a	Not detected	Not detected	Not detected	Not detected	Not detected	Not detected	**Detected**
**12a**	**Detected**	Not detected	Not detected	Not detected	Not detected	Not applicable	Not applicable
**12b**	**Detected**	Not detected	Not detected	Not detected	Not detected	Not applicable	Not applicable
**13a**	Not detected	Not detected	Not detected	Not detected	Not detected	Not detected	**Detected**
**13b**	Not detected	Not detected	Not detected	Not detected	Not detected	Not detected	**Detected**
14a	Not detected	Not detected	Not detected	Not detected	Not detected	**Detected**	**Detected**
15a	Not detected	Not detected	Not detected	Not detected	Not detected	**Detected**	Not detected

16a	Not detected	Not detected	Not detected	Not detected	Not detected	Not detected	**Detected**
**17a**	Not detected	Not detected	Not detected	Not detected	Not detected	Not detected	Not detected
**17b**	Not detected	Not detected	Not detected	Not detected	Not detected	Not detected	Not detected
18a	Not detected	Not detected	Not detected	Not detected	Not detected	**Detected**	**Detected**
19a	Not detected	Not detected	Not detected	Not detected	Not detected	Not detected	Not detected
20b	Not detected	Not detected	Not detected	Not detected	Not detected	**Detected**	Not detected
21a	Not detected	Not detected	Not detected	Not detected	**Detected**	Not detected	Not detected
22a	Not detected	Not detected	Not detected	Not detected	**Detected**	**Detected**	Not detected
23a	Not detected	Not detected	Not detected	Not detected	Not detected	Not detected	Not detected
24a	Not detected	Not detected	Not detected	Not detected	**Detected**	**Detected**	Not detected
25a	Not detected	Not detected	Not detected	Not detected	Not detected	**Detected**	Not detected
26b	Not detected	Not detected	Not detected	Not detected	Not detected	Not detected	Not detected
27a	Not detected	**Detected**	Not detected	Not detected	Not detected	Not applicable	Not applicable
28a	Not detected	Not detected	Not detected	Not detected	Not detected	Not detected	Not detected
29a	Not detected	Not detected	Not detected	Not detected	Not detected	Not detected	**Detected**
30a	Not detected	Not detected	Not detected	Not detected	Not detected	Not detected	Not detected
31a	Not detected	Not detected	Not detected	Not detected	Not detected	**Detected**	Not detected
32a	Not detected	Not detected	Not detected	Not detected	Not detected	Not detected	Not detected
33a	Not detected	Not detected	Not detected	Not detected	Not detected	**Detected**	Not detected
34a	Not detected	Not detected	Not detected	Not detected	Not detected	Not detected	Not detected
**35a**	Not detected	**Detected**	Not detected	Not detected	Not detected	Not applicable	Not applicable
**35b**	Not detected	**Detected**	Not detected	Not detected	Not detected	Not applicable	Not applicable
36a	Not detected	Not detected	Not detected	Not detected	Not detected	Not detected	Not detected
37a	Not detected	Not detected	Not detected	Not detected	Not detected	**Detected**	Not detected
38a	Not detected	Not detected	Not detected	Not detected	Not detected	**Detected**	**Detected**
**39a**	Not detected	Not detected	Not detected	Not detected	Not detected	Not detected	Not detected

**39b**	Not detected	Not detected	Not detected	Not detected	Not detected	Not detected	Not detected
40a	**Detected**	Not detected	Not detected	Not detected	Not detected	Not applicable	Not applicable
**41a**	Not detected	Not detected	Not detected	Not detected	Not detected	**Detected**	**Detected**
**41b**	Not detected	Not detected	Not detected	Not detected	Not detected	**Detected**	**Detected**
42a	Not detected	Not detected	Not detected	Not detected	Not detected	Not detected	Not detected
**43a**	Not detected	Not detected	Not detected	Not detected	Not detected	Not detected	Not detected
**43b**	Not detected	Not detected	Not detected	Not detected	Not detected	**Detected**	Not detected
44a	Not detected	**Detected**	Not detected	Not detected	Not detected	Not applicable	Not applicable
45a	Not detected	Not detected	Not detected	Not detected	**Detected**	Not detected	Not detected
46a	Not detected	Not detected	Not detected	Not detected	**Detected**	**Detected**	Not detected
47a	Not detected	Not detected	Not detected	Not detected	Not detected	Not detected	Not detected
48a	Not detected	Not detected	Not detected	Not detected	Not detected	Not detected	Not detected
**49a**	Not detected	Not detected	**Detected**	Not detected	Not detected	Not applicable	Not applicable
**49b**	Not detected	Not detected	Not detected	Not detected	Not detected	Not detected	Not detected
50a	Not detected	**Detected**	Not detected	Not detected	Not detected	Not applicable	Not applicable
51a	Not detected	Not detected	Not detected	Not detected	Not detected	Not detected	**Detected**
52a	**Detected**	Not detected	Not detected	Not detected	Not detected	Not applicable	Not applicable
53a	Not detected	Not detected	Not detected	Not detected	Not detected	**Detected**	**Detected**

"Detected" indicates that a cytogenetic or molecular (gene mutation/expression) marker is present; "Not detected" indicates that it is absent. Where one of the three markers is present that would stratify the patient into the cytogenetically favorable risk group i.e. inv(16)(p13q22)/t(16;16)(p13;q22), t(8;21)(q22;q22), or t(15;17)(q24;q21), the patient cannot be stratified into the intermediate risk group. Since in these cases the expression marker results are not applicable, the report output field reads "Not applicable" for these markers. "a" and "b" in sample ID represents bone marrow and peripheral blood respectively; sample numbers highlighted in bold indicate matched bone marrow and peripheral blood pairs.
